# Automatic lung segmentation in functional SPECT images using active shape models trained on reference lung shapes from CT

**DOI:** 10.1007/s12149-017-1223-y

**Published:** 2017-12-13

**Authors:** Grigorios-Aris Cheimariotis, Mariam Al-Mashat, Kostas Haris, Anthony H. Aletras, Jonas Jögi, Marika Bajc, Nicolaos Maglaveras, Einar Heiberg

**Affiliations:** 1 Laboratory of Computing, Medical Informatics and Biomedical-Imaging Technologies, School of Medicine, Aristotle University of Thessaloniki, Thessaloniki, Greece; 2 Department of Clinical Sciences Lund, Clinical Physiology, Skåne University Hospital, Lund University, Lund, Sweden; 3 Department of Biomedical Engineering, Faculty of Engineering, Lund University, Lund, Sweden; 4 Department of Clinical Physiology, Lund University Hospital, 22185 Lund, Sweden

**Keywords:** Image segmentation, V/P SPECT, CT, Active shape model

## Abstract

**Objective:**

Image segmentation is an essential step in quantifying the extent of reduced or absent lung function. The aim of this study is to develop and validate a new tool for automatic segmentation of lungs in ventilation and perfusion SPECT images and compare automatic and manual SPECT lung segmentations with reference computed tomography (CT) volumes.

**Methods:**

A total of 77 subjects (69 patients with obstructive lung disease, and 8 subjects without apparent perfusion of ventilation loss) performed low-dose CT followed by ventilation/perfusion (V/P) SPECT examination in a hybrid gamma camera system. In the training phase, lung shapes from the 57 anatomical low-dose CT images were used to construct two active shape models (right lung and left lung) which were then used for image segmentation. The algorithm was validated in 20 patients, comparing its results to reference delineation of corresponding CT images, and by comparing automatic segmentation to manual delineations in SPECT images.

**Results:**

The Dice coefficient between automatic SPECT delineations and manual SPECT delineations were 0.83 ± 0.04% for the right and 0.82 ± 0.05% for the left lung. There was statistically significant difference between reference volumes from CT and automatic delineations for the right (*R* = 0.53, *p* = 0.02) and left lung (*R* = 0.69, *p* < 0.001) in SPECT. There were similar observations when comparing reference volumes from CT and manual delineations in SPECT images, left lung (bias was − 10 ± 491, *R* = 0.60, *p* = 0.005) right lung (bias 36 ± 524 ml, *R* = 0.62, *p* = 0.004).

**Conclusion:**

Automated segmentation on SPECT images are on par with manual segmentation on SPECT images. Relative large volumetric differences between manual delineations of functional SPECT images and anatomical CT images confirms that lung segmentation of functional SPECT images is a challenging task. The current algorithm is a first step towards automatic quantification of wide range of measurements.

## Introduction

Ventilation/perfusion single photon emission computed tomography (V/P SPECT) is an important routine diagnostic tool, to validate pulmonary function in different diseases such as pulmonary embolism, pneumonia, heart failure and tumors. In V/P SPECT both a lung ventilation and perfusion scan is performed in direct succession. The recommended methods are based on the registration of ventilation and perfusion images to measure pulmonary function using quotient images. V/P SPECT methods have been validated on pigs, phantom, and in the clinic for diagnosis of pulmonary embolism by phenotyping character of ventilation/perfusion defects [1–3]. For phenotyping changes in pulmonary function, such as left heart failure, Jögi et al. developed a method to quantify perfusion gradient, which had been visually recognized since 1966 [4–6].

Segmentation is necessary to make objective measurements of lung function and characterization of pathophysiological changes. Previous work has been focused on semi-quantitative analysis [7–11]. User-independent segmentation of the lung is likely to be an essential further improvement to these techniques, to facilitate implementation on the larger scale for routine use. Quantitative segmentation is also the first step for the assessment of functional lung volumes.

Computed tomography (CT) is the reference method for lung morphology and lung volume estimation. For many clinical indications, ventilation and perfusion scintigraphy functional imaging is preferred and is the indicated method. To quantify functional changes, image segmentation is required as quantification methods need to quantify changes only inside the lung.

To date, there has been little focus on lung segmentation on V/P SPECT images [12]. He et al. derived the lung segmentation from V/P SPECT images by utilizing cross-entropy threshold selection. Kwa et al. [13] have focused on CT images and co-registration with SPECT images. For this segmentation process, the authors used a global binary segmentation method. A similar approach was used by Harris et al. [14], where CT images were registered to SPECT images to analyze lobar ventilation/perfusion relationships. Meir et al. [15] used registration of SPECT and CT images to perform texture analysis and demonstrated the potential to perform disease classification. Fleming et al. [16] described a method which uses empirical rules derived from the analysis of computer simulated images and local thresholds. The current aforementioned segmentation methods have not been validated against an independent reference standard.

The main challenge in lung SPECT segmentation is that there may be perfusion/ventilation defects so that the parts of the lungs are not possible to differentiate from the background and the use of thresholding is not sufficient to segment the lungs. To accurately delineate the lung contours, anatomical knowledge about the expected lung shape is required. The active shape model approach (ASM) is a method which incorporates the expected shape into the segmentation process, known as a priori information.

CT provides image contrast between lung tissue and surrounding tissue enabling anatomic lung contour to be more easily delineated. As such, CT also provides a suitable reference standard for the volume measurement of lung SPECT images.

The aim of the study was to develop an algorithm for automatic segmentation of the lungs in V/P SPECT images using active shape model of the lungs, trained by 3D shapes delineated in CT images and validate this automatic SPECT segmentation method against CT images as well as against SPECT manual delineations. Furthermore, the study compares manual segmentation in SPECT images against reference CT images so as to improve the understanding of limitations in segmenting V/P SPECT images.

## Methods

### Study population

In total 77 subjects were included. All subjects but eight come from a previously published study with known lung disease [10] The population were as follows; patients with stable chronic obstructive pulmonary disease (COPD) defined by spirometry (*n* = 55; age = 68 ± 5; 25 women; global obstructive lung disease score (GOLD) [1–4], smoking 15–159 pack-year), and a set of current or former long-time smokers (*n* = 14, age = 69 ± 3, 7 women, smoking 24–40 pack-year) that did not have COPD defined by spirometry. All subjects were over the age of 40 years, clinically stable and, in the case of COPD patients, without any exacerbations during the 4 weeks prior to inclusion. In addition to the above 69 subjects, 8 subjects were included retrospectively from clinical routine without apparent perfusion of ventilation loss in SPECT.

The study was approved by the local regional ethics committee, and informed consent was obtained from all subjects before enrolment. The subjects were imaged with a low-dose CT, in conjunction to the V/P SPECT examination.

The material was divided into two sets, one for training and one for performance evaluation. The evaluation set was chosen by randomly selecting 20 subjects from the population with known lung disease. In the evaluation set 12 of the selected subjects had COPD on spirometry and 8 where current or former smokers. The used random generator was Matlab R2014a (Mathworks, USA). The training set consisted of 57 subjects (the remaining 49 patients and 8 subjects). The purpose of a wide range of subjects from 8 apparently healthy subjects, 14 former or current smokers, and 55 with COPD was first to develop a robust algorithm that would be able to also handle both normal and challenging cases, and second investigate difference in manual lung segmentation in difficult cases.

### Imaging

The examination was performed as a one-day protocol, according to the European Guidelines [1, 17], where it is suggested that the ventilation is performed before the perfusion, followed by image acquisition.

V/P SPECT and low dose CT imaging were performed using a Philips Precedence system, which combines a dual head gamma camera with a Brilliance 16 slice CT. Imaging begun with a CT overview image and continued with a diagnostic low dose CT (120 kV, 20 mAs/slice, 16 × 1.5 collimator, 0.5 s rotation time, and pitch of 0.813). The slice thickness was 5 mm and incremental value was 5 mm. Filtered back projection was used for reconstruction. The CT was used to co-localize the morphological and functional changes visualized in either of the two modalities and obtained during free tidal breathing. Thereafter, the patients in supine position inhaled 30 MBq of Technegas, Cyclomedica Ltd and ventilation imaging was performed. This was followed by perfusion images were acquired using an intravenously injection of 140 MBq 99 m-Technetium labelled macroaggregated albumin (TechneScan LyoMAA; Mallinckrodt Medical BV). Care was taken to ensure that the patient maintained the same supine position. Imaging parameters were as follows, acquisition was performed in a 64·64 matrix, zoomed to a pixel size of 6.8 mm with 128 projections over 360°. Sixty-four steps, each of 10 s duration, were used for the ventilation study, and 64 steps of 5 s duration were used for the perfusion study. Reconstruction was performed using ordered subsets expectation maximization with eight subsets and two iterations.

## Segmentation algorithm overview

The segmentation was performed on a sum image of both ventilation and perfusion. The segmentation method was based on the active shape model approach [18]. In short, the active shape model approach a mean shape is place in the image and are allowed to deform until the shape fits the image. In a training step both mean shape as well as a priori information on allowed deformations are extracted.

**Fig. 1:**
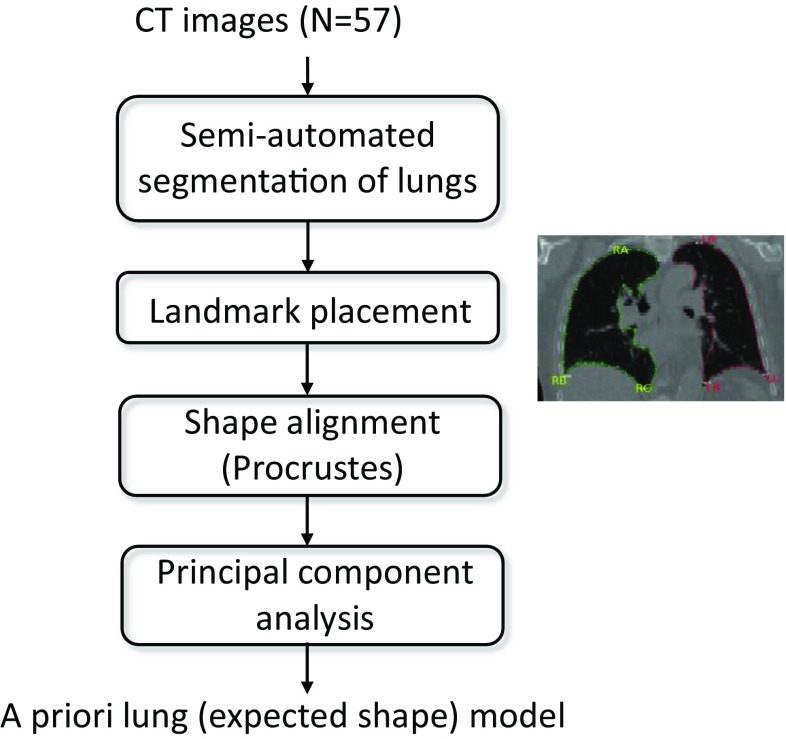
Flow-chart describing lung shape model creation. On the right, it is depicted the semi-automatic segmentation and the major landmark placement. The major landmarks are RA on top of the right lung, RB on the left of the right lung, RC on the right of the right lung, LA on top of the left lung, LB on the left of the left lung, LC on the right of the left lung

The training set was used to create two shape priori models, one for each lung. The training was based on lung shapes extracted from CT images in the training set. This training process was performed once and is illustrated in Fig. 1. More details are given in the section below.

## Shape model extraction

### Semi-automated segmentation of CT images

Contours in coronal CT slices were derived using an automated method that combined simple thresholding based on Hounsfield units and binary morphological operations to find the two largest coherent areas representing left and right lung, respectively. Thereafter further binary operations were performed to fill holes in the segmented lung shapes. Based on the edge of the binary objects, contours were extracted. The contours were carefully inspected by a trained observer (biomedical laboratory scientist), and were manually corrected to remove the vessels and other cardiac structures. For difficult cased another observer (expert physician) provided second opinion. An example of the semi-automated segmentation is illustrated in the insert of Fig. 1 which depicts the training process. Two sets of segmentations were constructed, one for each lung. As a consequence, the shape representation of the lung in CT images was a set of points distributed along the lung perimeter for each slice of the image volume.

The training phase consisted of four main parts: semi-automatic lung segmentation, shape parameterization, aligning shapes to each other and principal component analysis (PCA). The PCA computes orthogonal modes deformation based on all training examples. Training data consisted of semi-automatically segmented volumetric CT images. This information needed to be parameterized so as to permit usage of the active shape models theory. In the next step, the set of training shapes were aligned by a version of the Procrustes algorithm. In short, all points in a shape were transformed by stretching and rotating to best fit the mean shape. In the final part, PCA was performed and the outcome mean lung shape and its main variability modes were input to the segmentation procedure to constrain the model deformation to physiological plausible shapes.

The active shape model theory required that we mathematically described the lung in a way that for each point in one lung, we could identify the corresponding point in the other lung [19]. We used separate models for the left and right lung, respectively. Thus, there was a need for equal number of points in each lung. We selected three landmarks which were annotated for each lung in all imaging slices. These landmarks were defined as point extremas of slice contours: top, bottom left and bottom right. In between, $N$ points (pseudo-landmarks) of the original representation were sampled in equal distances [20]. As a consequence, every slice contour was described by $3 \times (N - 1)$ points, where 3 is the number of landmarks and *N* − 1 is the number of points between each landmark.

Having the same number of points in each slice is not enough we also require the same number of slices for each subject. Therefore, the training shapes were reshaped to keep a constant number of slices in the *z*- (from chest to back) direction. In the training set, the average number of slices were 30 (resolution in *z*-direction was 5 mm). We, therefore, chose to describe each shape with $3 \times (N - 1) \times 30$ points. The rationale for choosing 30 was to minimize the impact of slices resampling. The resampling procedure was carried out as follows: Each point in a slice was connected with a straight line to the corresponding point in the slices above and/or below. These connections resulted in 3 (*N* − 1) connected line segments. New points were interpolated on the intersections of these lines with a fixed number 30 slices with the same distance between them.

Thus, the representation of each lung *X* was $3 \times \left( {~N - 1} \right) \times 30$ points:

1 $$X=\left( {{x_{1,}}{y_1},{z_{1,}}..,{x_{n - 1}},{y_{n - 1,}}{z_{n - 1}},{x_{n,}}{y_{n,}}{z_n}} \right),$$

where *3* is the number of landmarks per slice, *(N-1)* is the number of points between landmarks, and 30 is the fixed number of slices.

### Alignment

To measure the shape variation, each lung *X* was transformed by translation parameters: ${t_x}$, ${t_y}$, ${t_z}$,scaling parameters *s*, and rotation parameters *θ, φ* into a common frame of reference.

2 $$X=~{T_{{t_x},{t_y},{t_z},s,\theta ,\varphi }}({\rm X}).$$

The centroid of a lung shape is defined by $C=\left( {{x_\mu },~{y_\mu },~{z_\mu }} \right)$, where ${x_\mu }$ is the mean value of ${x_1}, \ldots {x_{n - 1}},{x_n}$ coordinates of the shape (1) and so forth. By subtracting from each point of a shape its centroid’s coordinates $({x_\mu },~{y_\mu },~{z_\mu })$, the shape $X=\left( {{x_1} - {x_\mu },{y_1} - {y_\mu },{z_1} - {z_\mu },..,{x_{n - 1}} - {x_\mu },{y_{n - 1}} - {y_\mu },{z_{n - 1}} - {z_\mu },{x_n} - {x_\mu },{y_n} - {y_\mu },{z_n} - {z_\mu }} \right)$, is translated and its new centroid is the image origin (0,0,0). This process was repeated for all the shapes, so that all shapes had a common centroid.

Calculation of volume was defined by the Frobenius metric [12, 20]. The calculation was simplified due to the previous translation:

3 $${\text{Vol}}=~{\left( {\mathop \sum \limits_{i} \left( {{x_i}^{2}+{y_i}^{2}+{z_i}^{2}} \right)} \right)^{1/2}},$$

for all i points of a lung shape. Then, all coordinates were divided by ${\text{Vol}}$ so that all scaled shapes had volume |X| = 1 [20]. An implementation of the Procrustes algorithm rotated each shape so that the sum of distances $D=\mathop \sum \limits_{{i=1}}^{n} {\left| {{X_i} - \tilde {X}} \right|^2}$ of each shape ${X_i}$ to the mean shape $\tilde {X}=~\frac{1}{m} \times \left( {\mathop \sum \limits_{{i=1}}^{m} ~{X_{i~~}}} \right)$, was minimized (*m* is the number of training shapes).

Specifically, the following steps were performed: [20]. First, every shape was rotated to get aligned to one random shape of the set. Second, the mean shape was calculated and then every shape was rotated to align to it. This final step was repeated until a low value of sum of distances *D* was achieved (*D* < 1).

### Principal component analysis

The core of the shape model extraction was principal component analysis, which was used to define main variations of the shapes model.

To avoid non-linearity in the aligned training set, all shapes that were stored in one matrix were projected in tangent space by scaling them by $~{1 \mathord{\left/ {\vphantom {1 {\left( {{X_i} \times \tilde {X}} \right)}}} \right. \kern-0pt} {\left( {{X_i} \times \tilde {X}} \right)}}$ where $\tilde {X}~$ is the mean shape that occurred after the last iteration of the alignment process. The mean shape was subtracted from the shape matrix, to calculate the covariance matrix:

4 $$S=~\frac{1}{m} \times \left( {\sum\limits_{{i=1}}^{m} {\left( {{X_{i~~}} - \tilde {X}} \right) \times {{\left( {{X_{i~~}} - \tilde {X}} \right)}^{\rm T}}} } \right).$$

The eigenvectors $~{p_{i~~}}$ and eigenvalues $~{\lambda _{i~~}}$ of this covariance matrix were calculated. A shape instance could be generated by deforming the mean shape by a linear combination of eigenvectors:

5 $$\tilde {X}+P \times b,$$

where *P* was a matrix with *t* number of eigenvectors and *b* was the shape coefficients vector. The number of eigenvectors to retain, *t*, was chosen so that the model represented some proportion (e.g. 95%) of the total variance of the data [20]. Eigen modes that may be caused by outliers or by bad alignment were removed because they may have created unrealistic shapes. The selection of the number of eigen modes affected the quality of the shape model as well as the segmentation procedure. If the eigen modes were few then the segmentation was over constrained and if there were too many then the segmentation results may have appeared unrealistic.

## Segmentation

**Fig. 2:**
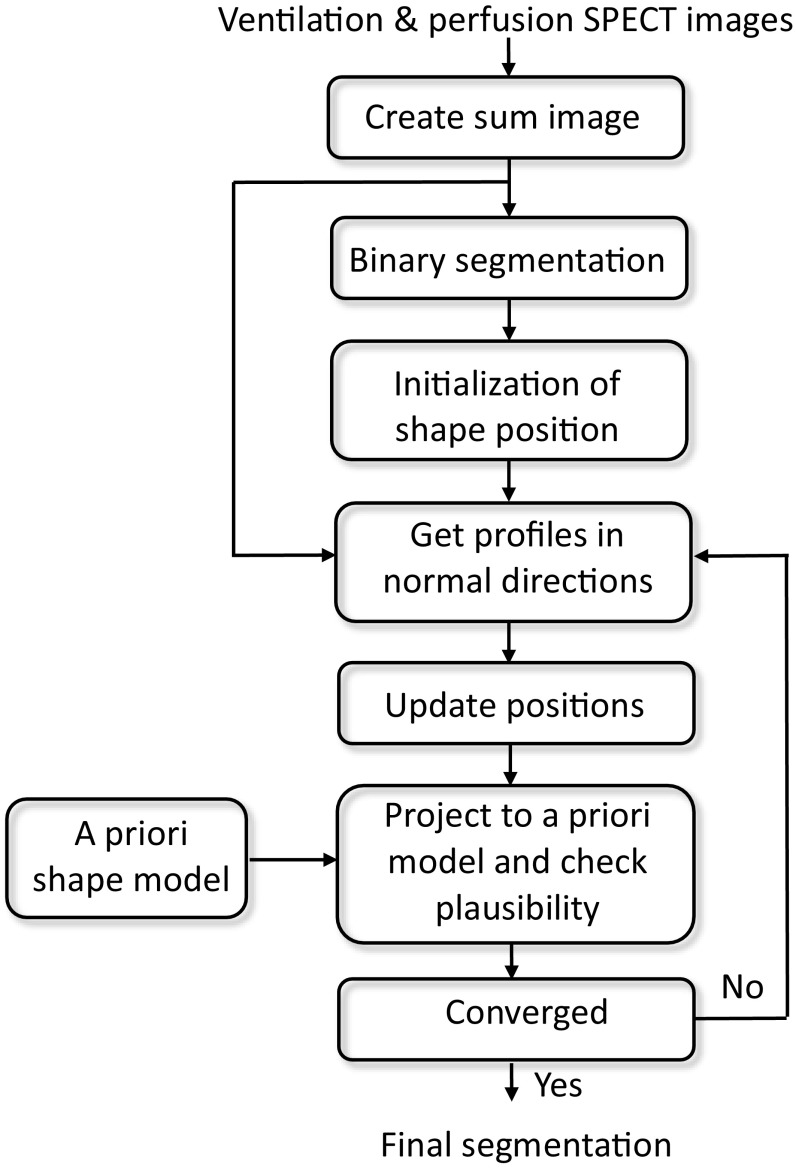
Flow-chart of the proposed automatic lung segmentation algorithm

A flow chart of the segmentation process is illustrated in Fig. 2. First, a summation of both ventilation and perfusion image volumes was created. Before summation, the two respective image volumes were normalized with the largest pixel intensity. The mean lung shape was placed into the image volume. The initial location was found by simple binary segmentation of the lungs. The lung shape iteratively updated both position and shape based on image intensities in both ventilation and perfusion images. The active shape model discarded shapes that were not plausible. This helped to overcome the possibility of erroneous segmentation due to matched defects and consequently helped to recognize such defects.

### Binary Segmentation

To identify initial location and size, a low threshold (15% of the maximum intensity of the image) was applied to extract a binary lung volume. This initial segmentation overestimated the volume, but was used to calculate the centroid of the lung. Furthermore, it allowed estimation of upper and lower limits of the lungs.

### Initialization-place shape

The initial shape was the mean shape calculated in the training phase. The shape was then transferred to fit the limits defined by binary segmentation. First, the mean shape was scaled to obtain the volume of binary shape. Then, it was rotated to be the reference shape. To align the mean shape $\tilde {x}$ to a shape of reference ${x_{{\text{ref}}}}$, their inner product ${X_p}$ was computed

6 $${X_p}=\tilde {X} \times X{~_{{\text{ref}}}}.$$

By performing single value decomposition on *X*, we get *U, S, V* i.e. the matrices of specific properties that satisfy the equation $X=U \times S \times {V^T}$. The rotated mean shape was calculated:

7 $${\tilde {X}_{{\text{rot}}}}~=(V^{\prime}*U^{\prime})*\tilde {X}^{\prime}.$$

Finally, ${\tilde {X}_{{\text{rot}}}}~$was translated so as to have the same centroid as the binary segmented volume.

### Update positions

The initial shape was iteratively updated according to intensity of pixels that were normal to the contour. For each update step, the shape was restricted to only generate contours that were plausible according to the training data [19]. Each point in the contour was moved to new positions based on intensity profiles and their derivatives which were normal to the contour. The location of the minimum of the derivatives indicated where to place the updated contour point. In addition, there are two rules that overrode the above behaviour so as to avoid getting stuck in local minima or maxima: (a) if the intensity on the contour point was above 30% then it moved outwards and (b) if the intensity was below 5% then it moved inwards (intensities were normalized in the space 0–1). This last rule was applied so as to ensure that the contour shrunk to the expected position if it extended outside the lungs. The shape was updated for a fix number of ten iterations which was after experiments was deemed sufficiently to achieve convergence.

### Constrain to plausible shapes

To ensure that the updated shape was plausible we first aligned it to the mean shape of the shape model [12]. Thereafter, we described the difference to the mean shape by projecting the shape on the shape model basis (i.e. we calculated $b=\Phi \times (X^{\prime} - \tilde {X})$, where *Φ* was the matrix with all eigenvectors that corresponded to the Eigen modes chosen). Then, if the elements in *b* were inside the space $\left[ { - {\lambda _i} \times \sqrt 2 ,{\lambda _i} \times \sqrt 2 } \right]$, where ${\lambda _i}$ the eigenvalue that corresponded to each *b*_*i*_ element, the movement calculated in boundary search was accepted. If not then their values were adjusted either to ${\lambda _i} \times \sqrt 2$ or$- {\lambda _i} \times \sqrt 2$. Therefore, either the original *b* parameters were kept or some were adjusted to the space $\left[ { - {\lambda _i} \times \sqrt 2 ,{\lambda _i} \times \sqrt 2 } \right]$. This process was iterated sufficiently for convergence (ten times).

## Validation

The semi-automated segmentation of the corresponding CT images was used as the reference standard. The accuracy of the automatic segmentation was evaluated both as difference in lung volumes compared to the reference CT standard, as well as comparison between automatic segmentation and manual segmentation in SPECT images using Dice coefficient.

To further evaluate the performance of the algorithm, the lung contour provided by the algorithm was compared with the manual SPECT delineation of the lung. Manual SPECT delineations were performed in a subset of 20 patients by one observer (biomedical laboratory scientist). For difficult cases another observer (physician) performed second opinion. The manual delineations were then compared to the automatic segmentation.

To validate accuracy of lung shape and position, the Dice coefficient was computed [21]. The Dice coefficient *D* is defined by $D=2 \times {{\left| {X \cap Y} \right|} \mathord{\left/ {\vphantom {{\left| {X \cap Y} \right|} {\left( {\left| X \right|+\left| Y \right|} \right)}}} \right. \kern-0pt} {\left( {\left| X \right|+\left| Y \right|} \right)}}$, where *X* represented all voxels of the reference and *Y* represented all voxels of the automated method. We also used sensitivity (*S*) and precision (*P*) over the entire image, defined as *S* = TP/(TP + FN) and *P* = TP/(TP + FP) with TP as the number of true positives (voxels that were part of both reference segmentation and automatic segmentation result), FP as the number of false positives (voxels that were segmented but were not part of the reference segmentation) and FN as the number of false negatives (voxels that were not segmented but were part of the reference). Separate analyses were made for the right and left lungs.

## Results

The mean values for reference volumes from CT, manual SPECT and automatic SPECT delineations for the right and left lung are presented in Table 1. The volumes from CT were 1673 ± 582 ml (left lung), and 2080 ± 633 ml (right lung). The volumes from automatic delineations were 1732 ± 403 ml (left lung), and 2085 ± 399 ml (right lung). The volumes from manual SPECT delineations was 1684 ± 505 ml (left lung), and 2044 ± 554 ml (right lung).

**Table 1:** The volumes for CT, manual delineations, automatic delineations and volumetric difference gives difference from reference CT volume (bias and SD)

	Left	Right
Reference volume CT (ml)	1673 ± 582	2080 ± 633
Automatic volume SPECT (ml)	1732 ± 403	2085 ± 399
Manual volume SPECT (ml)	1684 ± 505	2044 ± 554
Manual volumetric difference (mm^3^)	− 10 ± 491	36 ± 524
Automatic volumetric difference (mm^3^)	− 58 ± 420	− 5 ± 540

Negative numbers means larger volumes compared to reference CT volume. Results are given as mean ± standard deviation over 20 cases

**Fig. 3:**
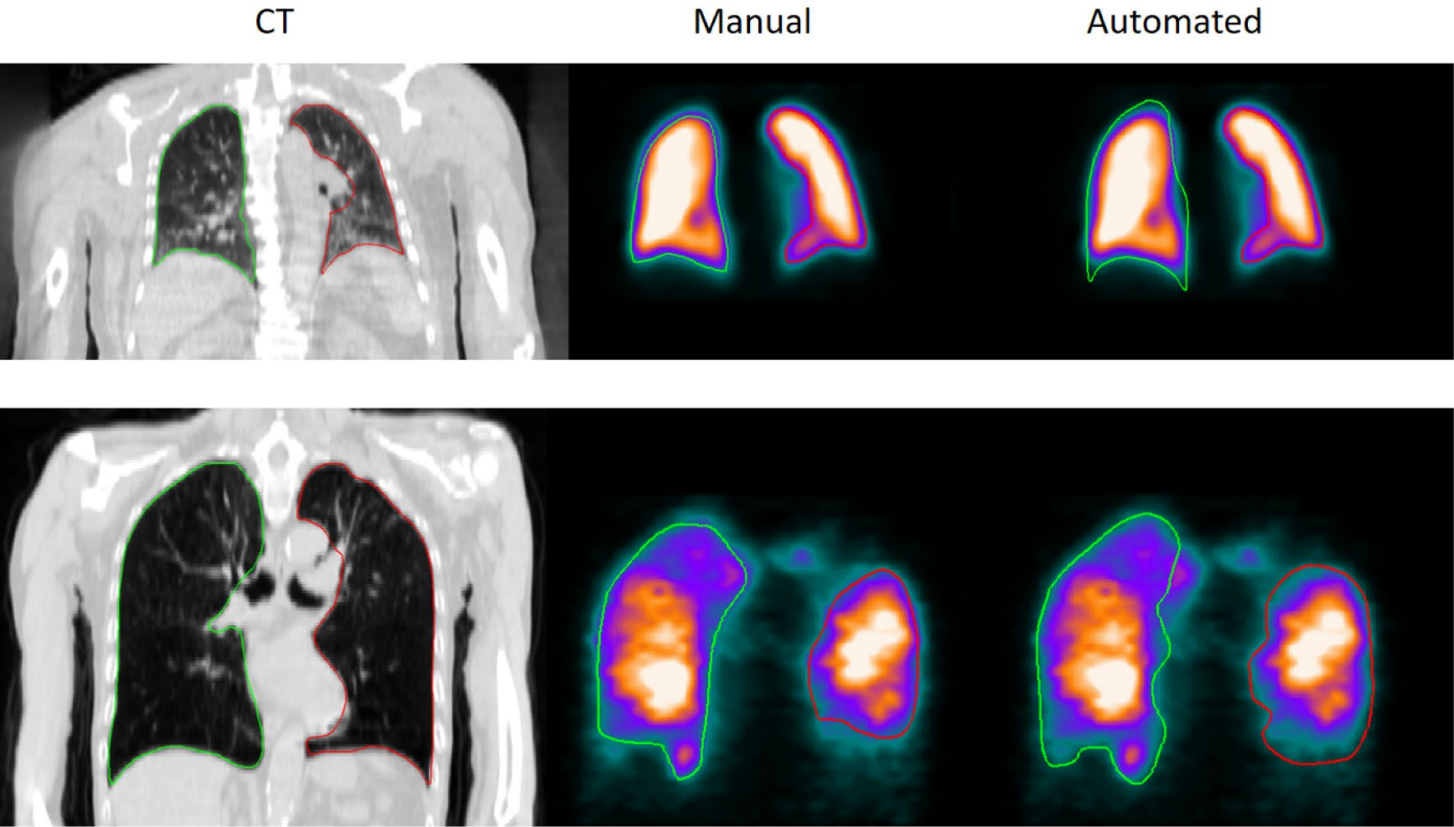
CT image, manual and automatic segmentation of two patients. Top row shows a slice from a patient with normal ventilation/perfusion and the bottom row shows a slice from a patient with peripheral loss of both ventilation and perfusion. The left column shows CT images, the middle column shows manual segmentations, and the right column shows automatic segmentations. Green delineation colour = left lung, red delineation colour = right lung

Figure 3 shows two patient examples, where to top row is a patient without any apparent loss of peripheral ventilation/perfusion and the bottom row shows a patient with peripheral loss of both ventilation and perfusion. Left column show CT image, middle column show manual segmentation, and right column show automatic segmentation.

### Comparison between manual SPECT and automatic SPECT delineations

**Fig. 4:**
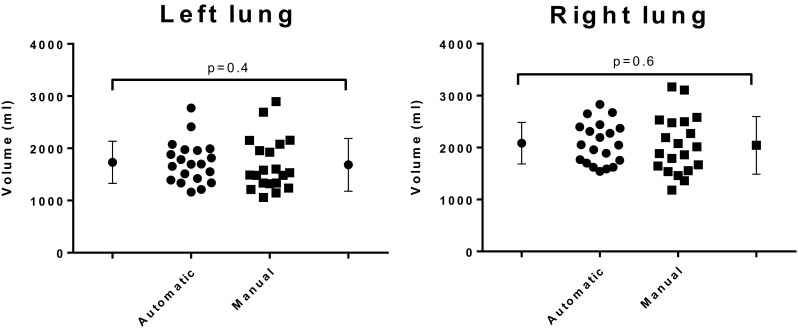
Comparison between automatic and manual delineations for the left and right lung

**Fig. 5:**
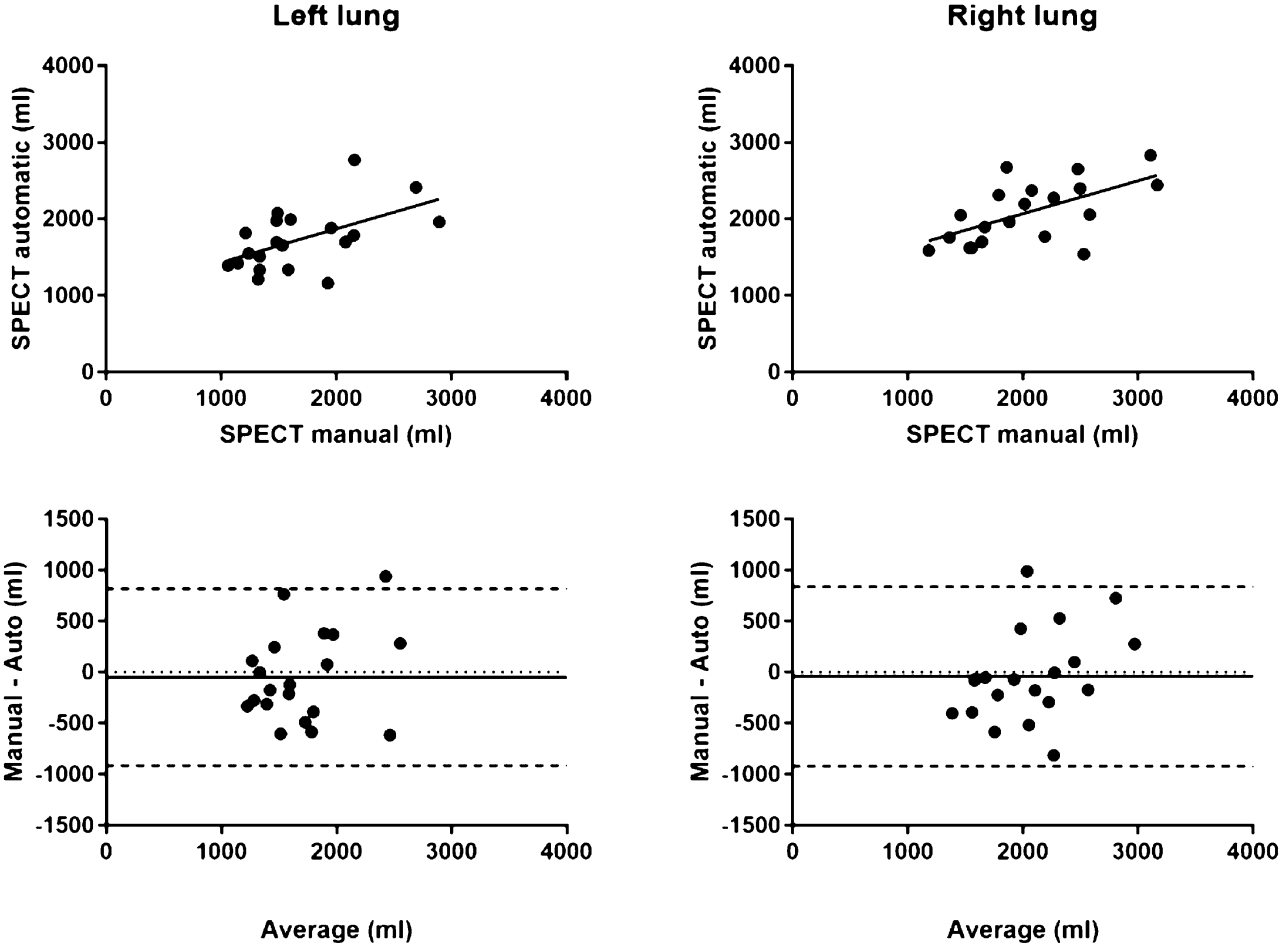
Scatter plot comparing manual and automatic segmentation on SPECT. Bottom panel, corresponding Bland–Altman plots for left and right lung, respectively

There was no observed statistically significant volumetric difference between manual SPECT and automatic SPECT delineations for the left and right, Fig. 4, *p* = 0.4 (left lung) and *p* = 0.6 (right lung). A summary of the comparison between manual SPECT and automatic SPECT delineations for the right and left lung are presented in Table 2. Figure 5 shows a scatter plot and a Bland–Altman plot comparing manual and automatic delineations in SPECT images.

**Table 2:** Comparison between automatic SPECT segmentation and manual SPECT delineation for the right and left lung

	Left lung (%)	Right lung (%)
Dice coefficient	82 ± 2	83 ± 3
Sensitivity	81 ± 9	82 ± 9
Precision	85 ± 9	88 ± 8

Results are given as mean ± standard deviation over 20 cases

### Automatic SPECT delineations vs reference CT

**Fig. 6:**
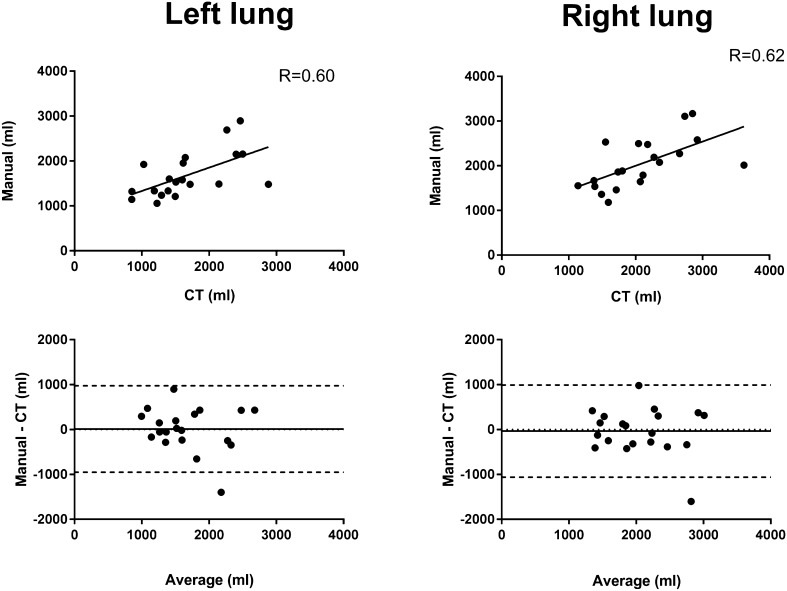
Automatic delineations vs CT for the right lung (right column) and left lung (left column). Upper panel: correlation plots for right and left lung. Lower panel: Bland–Altman plots

There was a significant difference between the reference volumes from CT and automatic SPECT delineations for the left lung (*R* = 0.69, *p* < 0.001, Fig. 6, left column) and right lung (*R* = 0.53, *p* = 0.02, Fig. 6, right column). For the left lung, the bias was − 59 ± 420 ml (Fig. 6, left column), and for the right lung, the bias was − 5 ± 540 ml, (Fig. 6, right column).

### Manual SPECT delineations vs reference CT

**Fig. 7:**
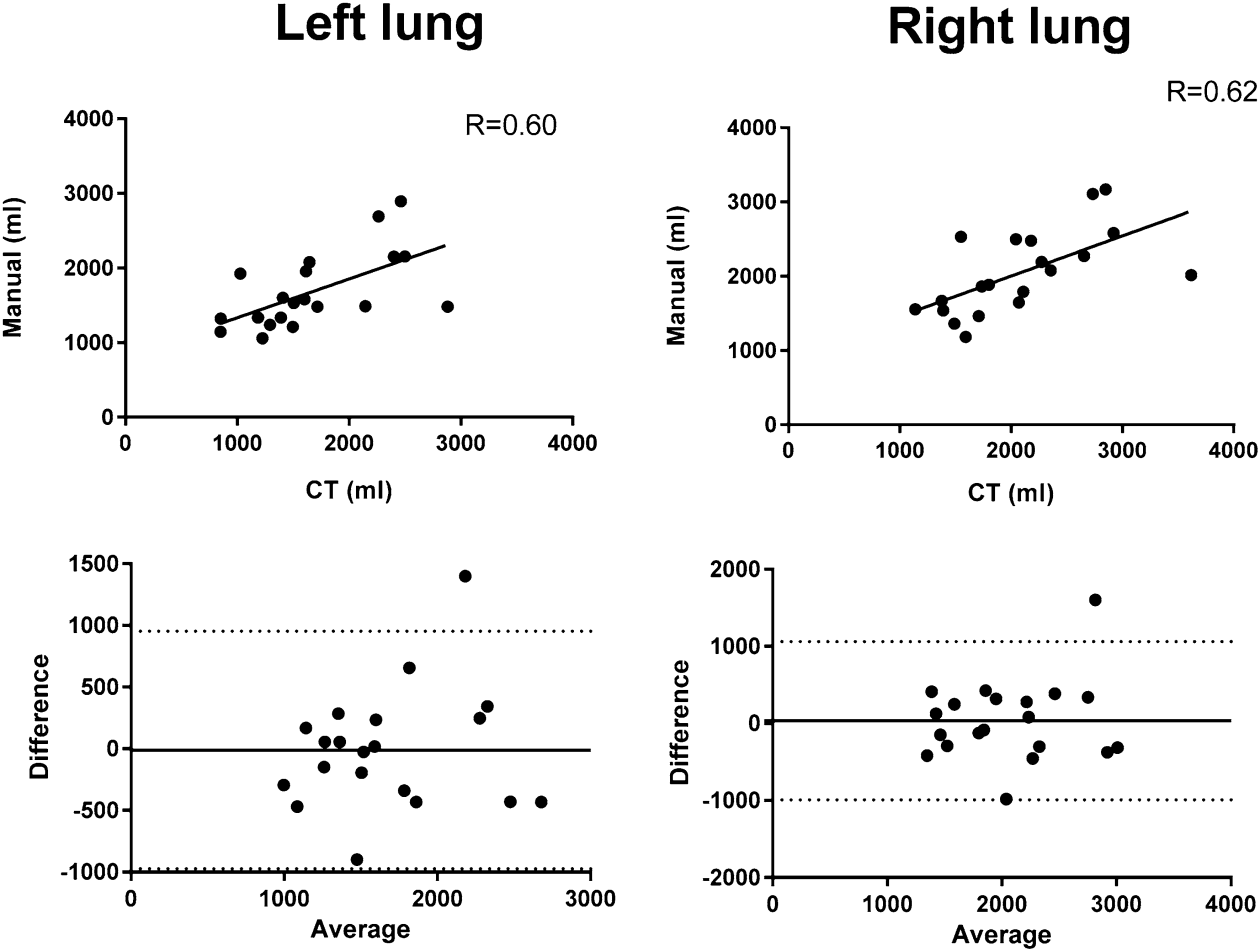
Manual delineations by observer 1 vs CT for the right lung (right column) and left lung (left column). Upper panel: correlation plots for right and left lung. Lower panel: Bland–Altman plots

There was a statistically significant difference between the reference volumes from CT and manual delineations on SPECT images for the left lung (*R* = 0.60, *p* = 0.005, Fig. 7, left column) and right lung (*R* = 0.62, *p* = 0.004, Fig. 7, right column). For the left lung, the bias was − 10 ± 491, (Fig. 7 left column). For the right lung, the bias was 36 ± 524 ml, (Fig. 7, right column). The coefficient of variation comparing manual SPECT delineation vs reference volume from CT are 29, and 25% for left and right lung, respectively.

## Discussion

A method for the automated segmentation of V/P SPECT images was developed. The automated segmentation and the manual delineations on V/P SPECT images yielded volumes that were not significantly different. The shape between automated segmentation and manual delineation on V/P SPECT images agreed well. However, both manual and automatic segmentation did not manage to estimate well with respect to the reference CT volumes. This highlights the difficulties in segmentation of lung volumes in functional images.

The idea of using anatomical lung shapes derived from reference CT lung images and use them to train an active shape model applied to lung SPECT images is new. The approach of cross imaging modality using active shape models were previously proposed by Ordas et al. where the authors presented an approach using active shape models extracted from cardiac magnetic resonance imaging for left-ventricle segmentation on SPECT images [22].

### Comparison between CT and SPECT images

The idea of comparing volumes from CT images with both automatic and manual delineations of V/P SPECT images is novel. CT images depicts the anatomical parts of the lungs, while the SPECT images depicts the functional parts of the lungs. Given this, this method illustrates the challenge of anatomical segmentation in functional SPECT images.

Importantly, there was no observed statistically difference between volumes extracted from manual SPECT and automatic SPECT delineations. The Dice analysis also confirmed that the results were similar and showed that the segmentation performance was better for the right lung compared to the left lung. This is in part expected, as the position and shape of the left lung is dependent on the position and shape of the heart, particularly in heart disease.

The results showed a significant difference between volumes from anatomical reference CT images compared to both automatic SPECT and manual SPECT delineations of functional images. One of the explanations is that the patients that were studied represent a group with very severe obstructive lung disease with large areas of reduced/absent ventilation and perfusion in the periphery. Peripheral loss of function limits the possibilities to find correct anatomical outlines by both manual SPECT and automated SPECT segmentations of the lungs.

### Image segmentation

The active shape model approach correctly estimated the expected lung shape regardless of loss of ventilation/perfusion. Peripheral defects still allowed the estimation of peripheral contour with the proposed automated segmentation method. An example is illustrated in Fig. 3.

### Limitations

We used CT as a reference standard for lung volumes. An alternative approach would be to use a lung phantom as an objective reference standard. The selection of landmarks is important for active shape appearance models. In this study, three landmarks (reference points in the lung contour) were used with a fixed number of contour points between each landmark (see landmark placement in Fig. 1). The landmarks worked well for the right lung. However, for the left lung it was observed that the landmarks LB and LC often were placed too close to each other and did not have a distinctive anatomical location. We tried to only use two landmarks (LA and LC), but this did not improve the results for the left lung.

### Future work

Given the results in this study with automated SPECT segmentation of the lungs it would be beneficial to add calculation of perfusion gradient and validate it to existing algorithms. Moreover, we would like to use an active shape model framework to automatically generate a 3D lung segment division. This could be accomplished by transferring manual lung segment charts [23] to the shape model. This would result in patient-specific lung segmental charts and could allow for the quantification of regional functional loss automatically.

## Conclusion

The algorithm presented in this study showed results comparable to manual delineations functional SPECT images. Relative large differences between manual delineations of functional SPECT images and anatomical CT images show that anatomical segmentation of V/P SPECT images is a challenging task. The present algorithm is a first step towards automatic quantification of wide range of measurements, such as perfusion gradients.
